# Multiple phases of human occupation in Southeast Arabia between 210,000 and 120,000 years ago

**DOI:** 10.1038/s41598-022-05617-w

**Published:** 2022-01-31

**Authors:** K. Bretzke, F. Preusser, S. Jasim, C. Miller, G. Preston, K. Raith, S. J. Underdown, A. Parton, A. G. Parker

**Affiliations:** 1 Department of Early Prehistory and Quaternary Ecology, University of Tübingen, Burgsteige 11, 72070 Tübingen, Germany; 2 The Role of Culture in Early Expansions of Humans Project, Heidelberg Academy of Science and Humanities, Heidelberg, Germany; 3 Seminar for Prehistoric and Protohistoric Archaeology, University of Jena, Löbdergraben 24a, 07743 Jena, Germany; 4 Institute of Earth and Environmental Sciences, University of Freiburg, Albertstr. 23b, 79104 Freiburg, Germany; 5 Sharjah Archaeology Authority, Government of Sharjah, Sharjah, United Arab Emirates; 6 Institute for Archaeological Sciences & Senckenberg Centre for Human Evolution and Palaeoenvironment, University of Tübingen, Tübingen, Germany; 7 SFF Centre for Early Sapiens Behaviour (SapienCE), University of Bergen, Øysteinsgate 3, Post Box 7805, 5020 Bergen, Norway; 8 Human Origins and Palaeoenvironments Research Group, School of Social Sciences, Oxford Brookes University, Gipsy Lane, Oxford, OX3 0BP UK; 9 Center for Microbial Ecology and Genomics, Department of Biochemistry, Genetics and Microbiology, University of Pretoria, Hatfield, South Africa; 10 Mansfield College, University of Oxford, Mansfield Road, Oxford, OX1 3TF UK; 11 ARC Centre of Excellence for Australian Biodiversity and Heritage, University of Wollongong, Wollongong, Australia

**Keywords:** Archaeology, Anthropology, Cultural evolution, Climate change, Palaeoclimate

## Abstract

Changing climatic conditions are thought to be a major control of human presence in Arabia during the Paleolithic. Whilst the Pleistocene archaeological record shows that periods of increased monsoon rainfall attracted human occupation and led to increased population densities, the impact of arid conditions on human populations in Arabia remains largely speculative. Here, we present data from Jebel Faya in Southeast (SE) Arabia, which document four periods of human occupation between *c.* 210,000 and 120,000 years ago. The Jebel Faya record indicates that human occupation of SE Arabia was more regular and not exclusively linked to major humid periods. Our data show that brief phases of increased rainfall additionally enabled human settlement in the Faya region. These results imply that the mosaic environments in SE Arabia have likely formed a population refugia at the end of the Middle and the beginning of the Late Pleistocene.

## Introduction

Presently a vast dry land, human occupation of Arabia is thought to be closely linked to climate change and limited to periods of increased precipitation^1–3^. Moisture is derived by both Mediterranean cyclones and through the African and Indian monsoon systems, in particular in the southern and western part of the peninsula^4–6^. The strength of the monsoon has varied through time due to orbital forcing^7,8^, with northern hemisphere glacial periods accompanied by low latitude aridity. Such periods of aridity are expected to represent hostile environments that formed an obstacle for human occupation of Arabia^9–11^. Increased precipitation occurred during past interglacials and interstadial periods as known from the mid-latitudes^9,12^, triggering the expansion of vegetation and increased faunal diversity in landscapes featuring lakes and perennial rivers in Arabia^13–15^. Due to the favorable living conditions during such periods, they are thought to be the most likely periods of intensified Paleolithic settlement in Arabia^14,16–18^.

The Arabian archaeological record supports the view of human occupation linked to periods of increased rainfall^19^. This is best exemplified by the coincidence of peak humid conditions between *c.* 130 and 75 ka and the well-developed archaeological records from many parts of the peninsula including Saudi Arabia^20–22^, the United Arab Emirates^23^ and Oman^24^. Additional evidence for human occupation of Arabia during wetter periods are known from the timeframe between 240 and 190 ka and between *c.* 60 and 50 ka and include sites in Saudi Arabia^25–27^, Yemen^28^ and Oman^29^. The archaeological record securely predating *c.* 240 ka is not well developed, but evidence from the Nefud (Saudi Arabia) points towards climate driven human occupation between 500 and 55 ka^19,30^.

Supposedly arid periods between *c.* 190 ka and 130 ka, *c.* 75 ka and 60 ka and *c.* 29 ka and 12 ka provide no evidence for human occupation of Arabia to date. Towards the end of the Pleistocene, the observed pattern of human occupation linked to wetter periods changes with sites from Saudi Arabia^31^ and Oman^32^ known to fall into the Pleistocene to Holocene transition at *c.* 12 ka.

Given available archaeological and paleoenvironmental records, linking increased humidity and increased human demography in Arabia is plausible. Due to the lack of data, the opposite, how increasing aridity affected human populations in Pleistocene Arabia, remains largely speculative. Researchers argue on the one hand that the lack of archaeological evidence for human occupation of Arabia in dry periods is linked to pan-Arabian extinctions and the abandonment of settlement in Arabia^19^. While other researchers argue that desiccation led to the contraction of human populations into refugia such as the Gulf basin region, the Dhofar Mountains and adjacent littoral zone as well as the Red Sea coastal plain^33^. Both explanations are mainly based on untested ecological hypotheses. Fossil and artifactual evidence are difficult to record, given large parts of the refugial zones are today below water, while current paleoclimatic records fail to adequately reflect the complexity and heterogeneity of the contemporary Arabian landscape.

**Figure 1:**
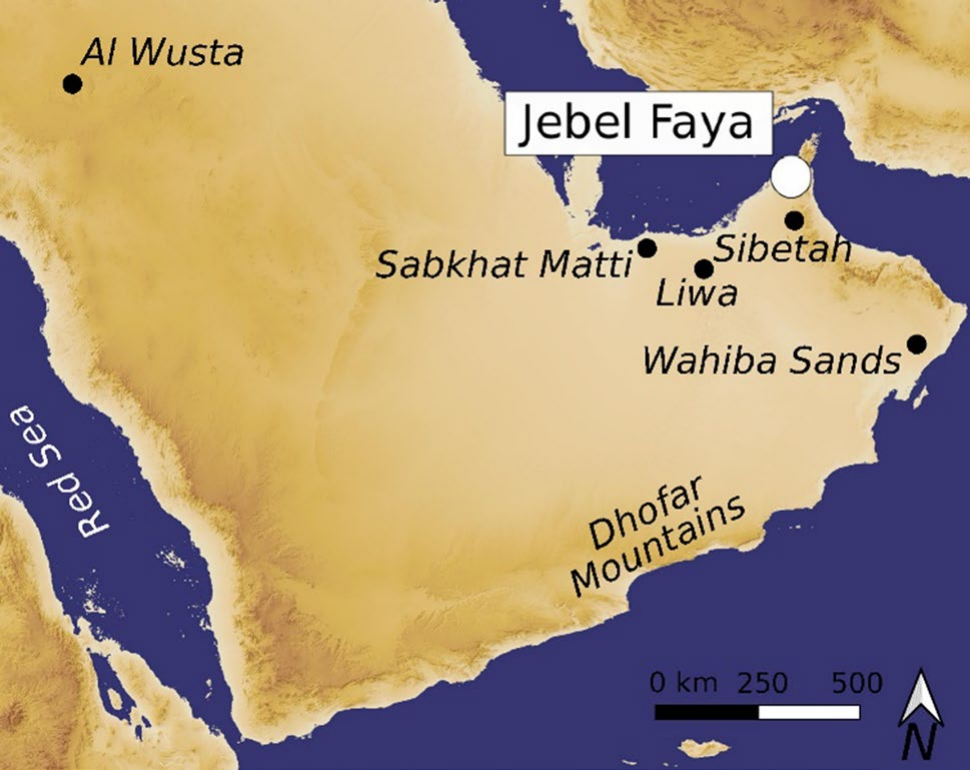
Map showing the location of Jebel Faya in Southeast Arabia and other sites and geographic regions mentioned in the text. Figure produced using QGIS 3.4. (https://qgis.org) and Inkscape 1.0 (URL: https://inkscape.org).

**Figure 2:**
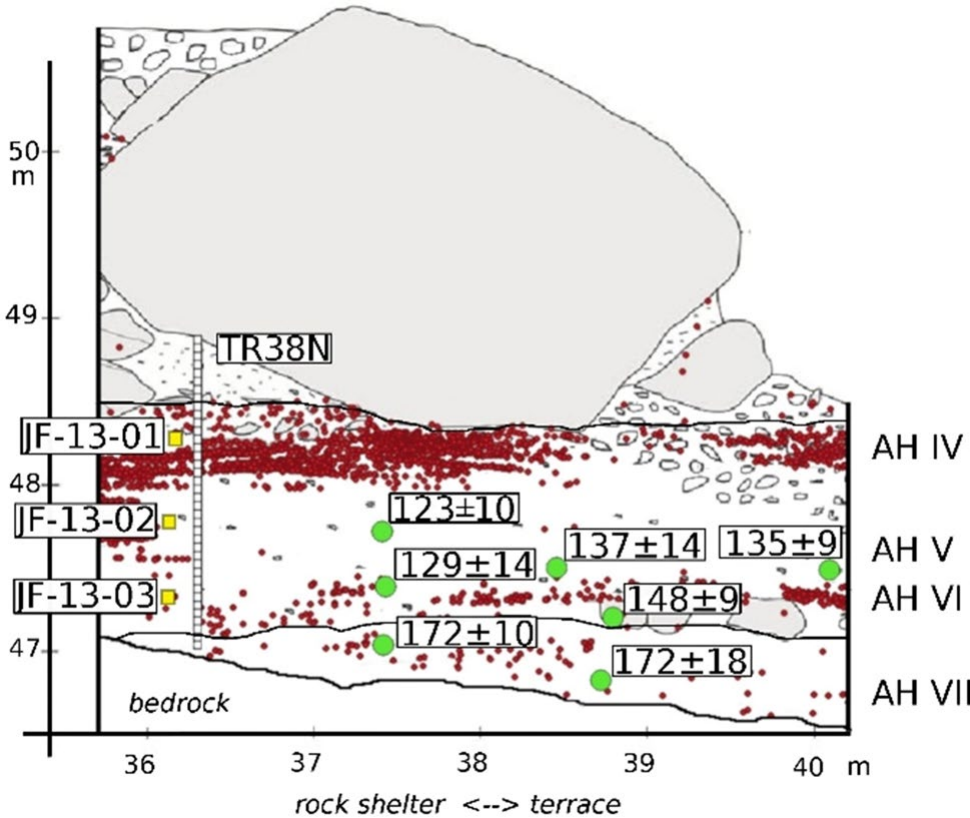
Northern profile of trench 38 at Jebel Faya showing the location of the lithic artifacts from AHs VII-IV (red dots), the location and results of the OSL samples presented here (green circles) as well as the samples for the sedimentological (white squares) and micromorphological study (yellow squares).

To study the impact of arid conditions on Paleolithic human populations in Arabia, long and securely dated archaeological sequences are required. Sites providing such characteristics are rare. One such place is Jebel Faya in the Emirate of Sharjah, United Arab Emirates (Figs. 1, S1), where a Paleolithic sequence with four archaeological assemblages A-D was excavated in front of a rock shelter^23^. While assemblage D, the lowermost archaeological layer of this terrace sequence, remained undated, the oldest age of around 125 ka was determined for Assemblage C^23^. Assemblage A from the top of the Paleolithic sequence on the terrace was dated to about 40 ka^23^. Recent field work at Jebel Faya focused on the sequence excavated within the rock shelter area. Excavations exposed a *c.* 3 m deep sequence with seven archaeological layers (AHs I-VII). Researchers were able to link the rock shelter sequence stratigraphically to the terrace sequence^34^. This shows that the dated Assemblages C and A from the terrace correlate with AHs VI and IV from the rock shelter. One key characteristic of the rock shelter sequence, however, is that it extends the known archaeological stratigraphy from previously unknown Middle Pleistocene artifacts (Figs. 2, S2). Here, we present new archaeological, chronological and sedimentological data from Jebel Faya that cover the Middle to Late Pleistocene transition.

## Results

**Figure 3:**
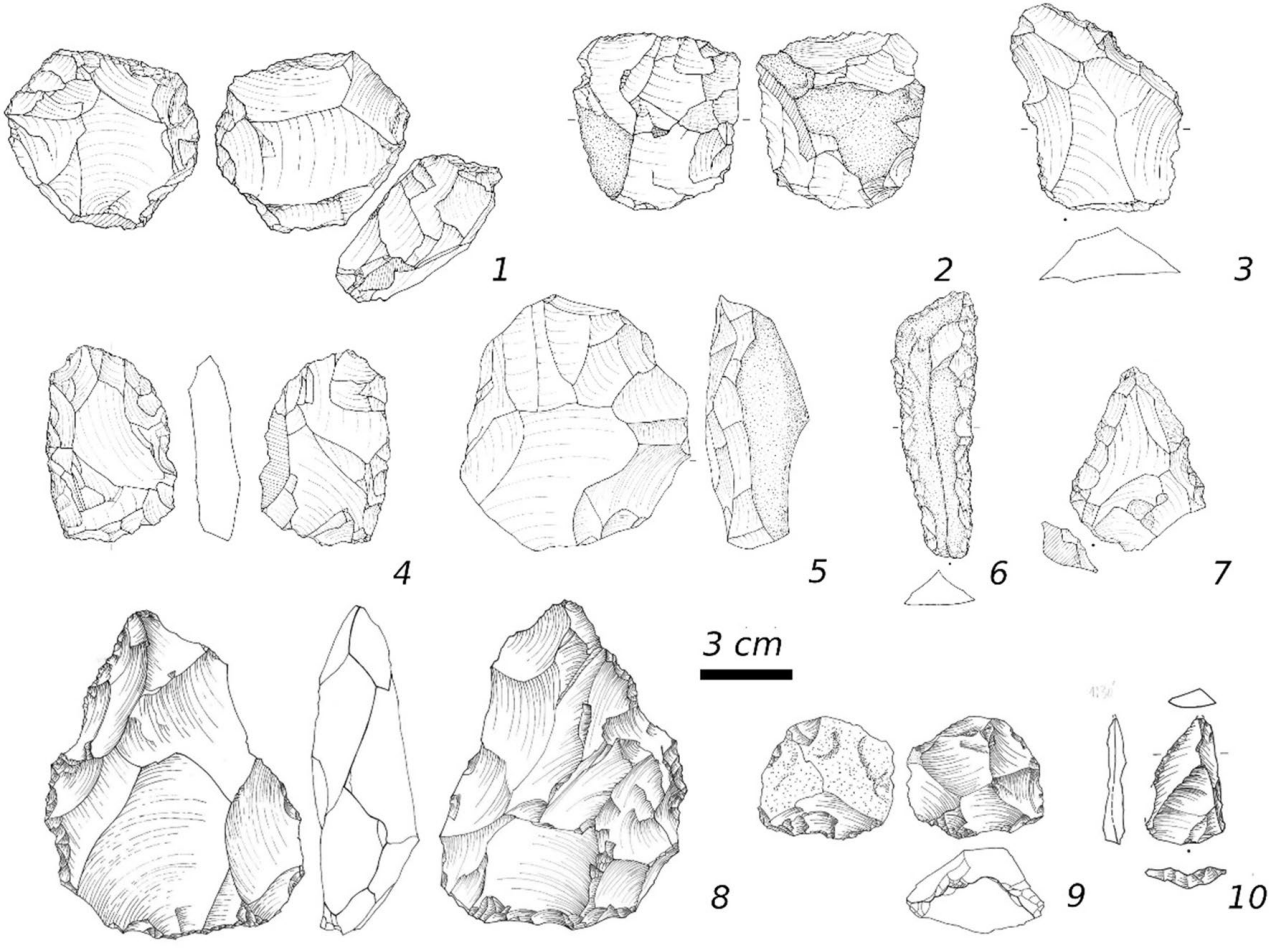
Examples of stone artifacts from AH V (**1**–**3**), AH VI (**4**–**7**) and AH VII (**8**–**10**). (**1**) inversely retouched scraper, (**2**) unidirectional Levallois core, (**3**) sidescraper, (**4**) bifacial artifact, (**5**) centripetal Levallois core, (**6**) retouched blade, (**7**) point, (**8**) bifacial artifact, (**9**) centripetal Levallois core, (**10**) point.

Excavations of the rock shelter (see methods and supplement for details) led to the recovery of an intact sequence of seven archaeological horizons (AHs). We focus here on the lower half of the Faya rock shelter sequence with AHs VII, VI and V (Fig. 2). In general, the archaeological assemblages from AHs VII to V feature a typical mix of Middle Paleolithic technologies and tool types (Fig. 3, Tables S1–S3).

The oldest archaeological layer from the base of the sequence is AH VII, which provides a lithic artifact assemblage (n = 301) that shows evidence for flake production from centripetal and unidirectional Levallois cores (Fig. 3, Table S3). Faceting of striking platforms and a bifacial component are additional technological features observed in AH VII (see Table S3 for details). The tool assemblage is dominated by side scrapers and denticulated artifacts but points and bifacially retouched artifacts do also occur (Fig. 3). Optically Stimulated Luminescence (OSL) dating of two sediment samples (FAYA-38S-OSL5: 172 ± 10 ka, FAYA-38S-OSL6: 172 ± 18 ka) provide for AH VII a mean burial age of 172 ± 9 ka (Table S4).

About 20 cm above AH VII, the next younger archaeological layer AH VI provides an assemblage of lithic artifacts (n = 477) dominated by centripetal and unidirectional Levallois reduction accompanied by bifacial reduction and blade production (Table S2, S3). The tool assemblage includes bifacial pieces, side scrapers, and denticulated flakes (Fig. 3). OSL sesults from within layer AH VI (FAYA-38S-OSL7: 129 ± 14 ka, FAYA-38S-OSL 4: 137 ± 14 ka, FAYA19: 135 ± 9 ka) reveal a mean depositional age of 134 ± 7 ka and an age of 148 ± 9 ka (FAYA-38S-OSL8) for the archaeologically sterile layer below. Furthermore, 10 cm above AH VI, a layer of lithic artifacts (n = 755) forms AH V. The lithic assemblage provides evidence for flake production from flat cores featuring convergent and orthogonal scars as well as from unidirectional Levallois cores (Table S3). An increased proportion of lithic artifacts featuring converging edges is evident in this layer, but there are no indications for true converging flaking technologies (see SI for details and^34^). Scrapers and denticulates form the majority of the tool assemblage accompanied by an increased number of pointed artefacts (Fig. 3, Table S2). The OSL sample from AH V produced a result of 123 ± 10 ka (FAYA-38S-OSL3).

Sedimentological and stratigraphic characteristics have been considered during excavation (see supplement). To gain more detailed insight into site taphonomy additional analyses were conducted in the 2 m thick section TR38N (Fig. S3) and from three micromorphology samples (JF-13-1, JF-13-2, JF-13-3). These samples are located under a massive rock shelter roof collapse deposit, which contains the lower part of the Faya rock shelter sequence comprising archaeological layers AH VII to AH IV (Fig. 2). Our results show that occupation layers tend to be dominated by sediments with an exogenic rock shelter component and an aeolian component (AD facies, see methods). In contrast, the archaeologically sterile layers tend to be dominated by facies originating from infiltration processes (facies FO) that commonly contain debris and rock fragments derived from weathering into finer grained material^35^. Further it has to be noted that host limestone bedrock comprises < 1% sand, and that it is emplaced due to gravity and rockfall processes. Beds dominated by rockfall debris (facies RD) also occur. They often show large blocks up to 50 cm derived from the collapse of part of the ceiling of the shelter with interstitial matrix derived from infiltration processes. Micromorphological analysis of the Faya rock shelter sequence confirms the interpretation of a largely aeolian source of sediment for the fine fraction of AH V and IV. Post-depositional alteration of the deposits is present, although weak. Evidence for localized carbonate cementation in the form of interstitial growths of micrite is present, as is localized iron and manganese staining of the fine fraction. There are also rare, infilled burrows, but despite a general absence of sedimentary structures, bioturbation does not appear to have significantly impacted the deposits.

Additional chronometric data has been collected from the lowermost archaeological layer in the Faya terrace sequence (Fig. S2). Assemblage D has previously not been dated. The lithic assemblage from this layer is relatively small (n = 177) and hence difficult to interpret beyond the notion of human presence at the site. Our OSL sample (FAYA-19N-OSL9) from sediments containing assemblage D indicates a burial age of the lithic assemblage of 212 ± 19 ka.

## Discussion

The lower part of the Jebel Faya rock shelter record presented contains a sequence of Middle Paleolithic assemblages, which show human presence at about 210 ka, 170 ka, and between 135 and 120 ka. These new data deepens the occupation history of the site substantially and identify previously unknown phases of human occupation in Southeast (SE) Arabia pre-dating Marine Isotope Stage 5 (*c*. 130–75 ka). Our data reveals that human occupation was more regular and not restricted to prolonged periods of increased rainfall. Instead, it indicates that brief wet spells may have played an important role on shaping human presence in SE Arabia.

OSL results from the bottom of the sequence place AH VII firmly into early MIS 6. Dating of AHs VI and V shows that the lithic assemblages have been most likely discarded at the beginning of MIS 5. While saying this, our results do not exclude the possibility of AH VI representing human occupation at the end of MIS 6. Given a c. 25 ka year gap between AHs VII and VI the typological and technological similarities of the lithic assemblages are striking. Rather simple tool types and minor shifts in the preference for the tool production systems suggest that no sophisticated adaptation in the lithic culture was required to survive in the ecological settings of the Faya region during the late Middle and early Late Pleistocene.

The new chronometric data from the Faya rock shelter sequence corresponds well with previous results presented for Faya^23^. Age estimations for AH VI (mean depositional age: 127–141 ka) fall within the range of the stratigraphically linked Assemblage C, for which Armitage et al.^23^ provide age estimations of 111–143 ka and 113–133 ka. While stratigraphic order would dictate that AH V is younger than AH VI, our OSL results indicate that AH V was deposited between 113 and 133 ka ago. The age of AHs VI and V are thus statistically indistinguishable. Assemblage B, overlaying Assemblage C in the terrace excavation, is stratigraphically linked to AH V. Since Assemblage B is undated, it provides no information that could help regarding the question of the chronological gap between AHs VI and V. Both excavations (terrace and rock shelter), however, similarly described an only small depositional buffer between the two archaeological layers of about 10 cm. It could thus be assumed that the chronological gap between AH VI and V as well as Assemblage C and B is relatively short. Age estimates of about 139–157 ka for sediments below AH VI and of 163–181 ka (mean depositional age) for AH VII indicate stratigraphic integrity of the rock shelter sequence and the robustness of the presented chronological framework.

The Pleistocene human occupation at Jebel Faya has previously been thought to be restricted to long periods of increased rainfall, in particular during MIS 5^23,36^. This conclusion is largely drawn indirectly from speleothem records from Hoti Cave in the Hajar Mountains and Al-Mukalla Cave in Yemen, which demonstrate speleothem growth in MIS 7 and MIS 5^37,38^. The onset and termination of MIS 6 are recorded in the Hoti and Al Mukalla speleothem records, but they do not extend into MIS 6. Given that the formation of speleothems is determined by rainfall, it has been suggested that precipitation in excess of 350 mm per year is required to initiate growth (e.g.,^38,39^). Paleoenvironmental evidence from SE Arabia indicates that a relatively small amount of rainfall was sufficient to activate alluvial fans and generate fluvial channel flow in MIS 6^40,41^ but was insufficient for speleothem growth. The Jebel Faya records testify that local geographic and environmental conditions can support a more frequent human occupation in SE Arabia beyond global peak interglacial periods. This reflects the importance of producing spatially finer scales of analyses, instead of linking local archaeological records with global climate proxies (e.g.,^2,42^).

**Figure 4:**
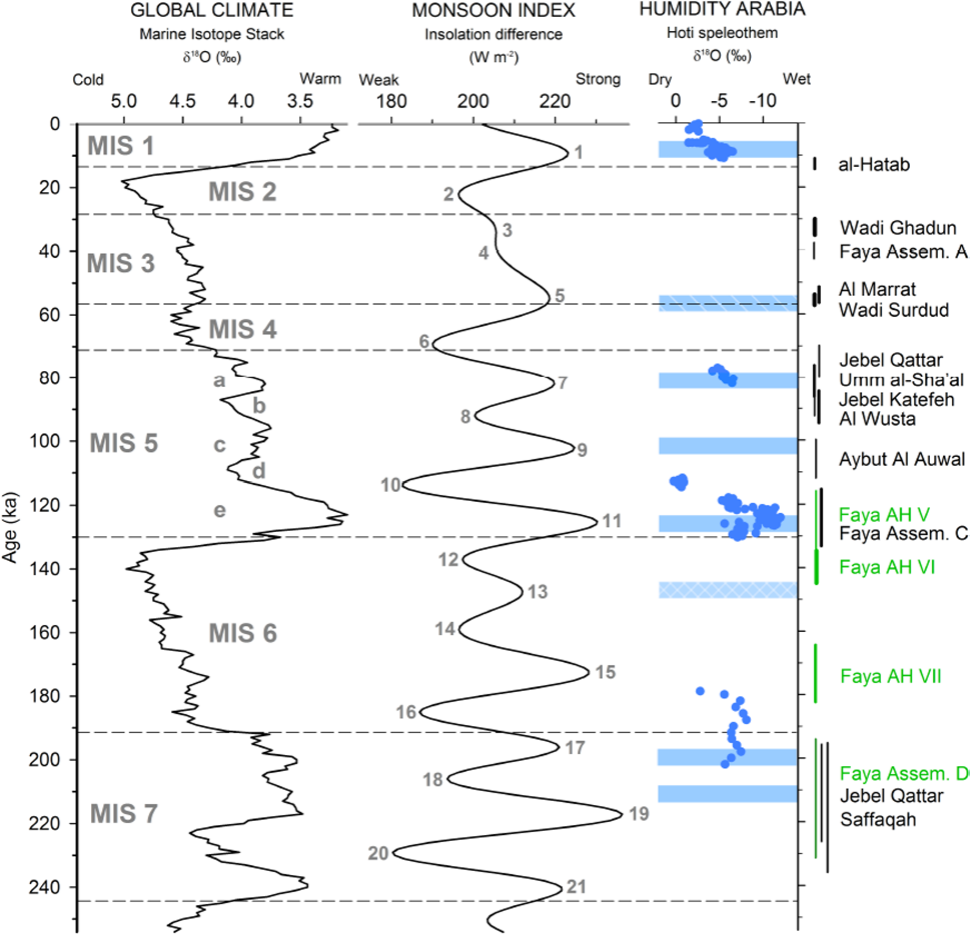
Marine isotope stack as proxy of global climate^56^, the Monsoon Index^43^, speleothem isotope data (blue circles^37^) as well as phases of lake formation (blue bars^4,6,11,13–15,41,45,47^) as representation of regional environmental conditions in Arabia. Note the co-occurrence of odd numbered Monsoon Index Peaks (MIP) and the archaeological record. Al-Hatab^32^, Wadi Ghadun^29^, Faya Assemblage A + C^23^, Al Marrat^57^, Wadi Surdud^28^, Jebel Qattar + Jebel Katefeh^25^, Umm al-Sha’al^27^, Al-Wusta^20^, Aybut Al Auwal^24^, Faya AH V–VII + Faya Assemblage D (this paper), Saffaqah^26^.

The traditional approach would contextualize the Faya archaeological record with MIS 7, 6 and 5. However, the link between global marine isotope records and regional climates is not straightforward and confirming this link with local records is difficult, given environmental evidence is poorly constrained and age control is often problematic. We argue that the Amero-Eurocentric (glacial vs. interglacial) view on the climatic evolution in Arabia does not fit well with the evidence that has been collected in the field over the past 20 years. Instead of using global marine data, we see more promise in a regional climate approach. In the absence of better alternatives at present, we suggest using the Indian Ocean Monsoon (IOM) Index to model the timing of favorable conditions for human occupation in Arabia. The IOM Index reflects precessionally-driven changes in monsoon intensity, based on latitudinal insolation differences^7,43^. This theoretical approach has been confirmed by field evidence, showing that increased precipitation in Arabia corresponds with maxima in the monsoon intensity^17,44,45^. The timing and frequency of the Monsoon Index Peaks (MIP) increases the chronological resolution for some parts of the Middle to Late Pleistocene history compared to the *c.* 100 ka eccentricity driven periodicity of the prevailing glacial-interglacial model (Fig. 4). The chronology of empirical evidence for human occupation in Arabia corresponds well with odd numbered MIPs (Fig. 4), which represent increased insolation driven monsoon intensity as a result of greater seasonality and increased pressure gradients across the Indian Ocean. The correlation of archaeological evidence and MIPs can be demonstrated for the last 130 ka (Fig. 4). However, this link is currently difficult to assess for the Middle Pleistocene to date due to the scarcity of archaeological and paleoenvironmental sites in Arabia. The timing of AH VII and Assemblage D provided here, contribute to the filling of these gaps, and indicates that a correlation of human presence and increased monsoon intensity existed also during the Middle Pleistocene.

The IOM Index indicates two phases of increased humidity within MIS 6 (MIPs 15 and 13, see Fig. 4). This shows that emphasizing exclusively arid or hyper-arid conditions for the *c.* 60 ka period of MIS 6 would oversimplify what is a particularly complex situation. This conclusion corresponds with previous claims for brief wet phases in MIS 6^1,41,46^. While wet phases have been identified in the otherwise arid MIS 6, human presence in Arabia during this period was unknown. AH VII now fills this gap and provides evidence for human occupation of SE Arabia in early MIS 6. The deposition of AH VII correlates with MIP 15, which reaches an intensity similar to the range of MIP 11 (Fig. 4). The latter represents MIS 5e, a period where a well-developed paleoenvironmental record shows pronounced and spatially wide-spread favorable conditions, including the development of perennial lakes and rivers^13–15,47^. Given currently available data, it is difficult to determine if conditions during MIP 15 (AH VII) were indeed as favorable as in MIP 11. Given the Faya evidence, however, it is reasonable to conclude that at the very least, climatic conditions and resource availability were sufficient for a human population to survive for some time in SE Arabia during early MIS 6. While AH V correlates well with MIP 11 and AH VI falls into the period of decreasing monsoon intensity following MIP 13, the correlation of Assemblage D with one MIP is not straightforward due to dating uncertainties. It may either be associated with MIP 19 or MIP 17.

Our archaeological data adds to a growing body of paleoenvironmental evidence that indicates brief phases of increased precipitation occurred during MIS 6. Evidence for increased water availability in the Faya region during MIS 6 comes from U series dating of carbonate precipitates (*c.* 168–161 ka and *c.* 146–136 ka) at Jebel Buhais, about 15 km south of Faya^48^. Elsewhere in SE Arabia, the prospect of increased rainfall during MIS 6 is corroborated by OSL dating of fluvial silts (*c.* 147 ka) at Sabkhat Matti^40^ and interdunal sabkha sediments in the Liwa region of the Rub’ al Khali, UAE, dated between 176 and 144 ka^49^. Situated within an alluvial plain that extends *c.* 500 km along the western flanks of the Hajar Mountains, alluvial fan sediments from the Al Sibetah site, UAE (Fig. 1) record over 20 m of sediment accumulation during MIS 6 between 179 and 130 ka^41^. Evidence from Al Sibetah shows that during this time braided streams surrounded by a vegetated landscape comprising a mix of C_3_ and C_4_ grassland types extended more than 20 km from the mountain front. At the southern end of the Hajar, incipient soil formation within the Al Jabin Unit of the Wahiba Sands region have been dated between *c.* 160 and 140 ka^10,50^. In sum, paleoenvironmental and archaeological records show that MIS 6 in SE Arabia was not a permanently hyper-arid period and human occupation was possible during that time due to increased rainfall and the development of vegetation in brief phases corresponding with at least MIP 15, but also likely MIP 13.

The lower half of the Faya record shows that human populations have occupied SE Arabia more frequently than previously recognized, while the significant chronological gaps between the occupation phases at Jebel Faya indicate repeated re-occupation of the site. Human presence at Faya during a relatively short-lived wet phase such as MIP 15 (AH VII), however, suggests a relatively rapid re-occupation, which would likely be linked to a spatially neighboring source region rather than long distance movements.

Presently SE Arabia is characterised by steep rainfall gradients across a range of coastal, mountainous, desert and gravel plain geomorphic environments, giving rise to a diverse range of biogeographical distributions and centers of endemism. In particular, the Western Hajar Mountains represent one of the richest and most important floristic regions within the Arabian Peninsula, being home to 24 endemic plants species including evergreen communities indicative of relict former xeromorphic woodlands^51^, and three species of freshwater fish found within mountainous wadis and pools^52^. Consequently, we suggest that rainfall increases during MIS 6 would have been sufficient to activate widespread drainage and increase floral and faunal productivity within the Faya region. Our results demonstrate an increased number of periods where inland areas of SE Arabia provided favorable environmental conditions and attracted human occupation.

From where exactly the Faya populations geographically originated remains unknown, but given indications of rapid re-occupation, shared technological characteristics and biogeographic characteristics, we conclude that our results support the idea of a population refugia in SE Arabia at the end of the Middle Pleistocene and the beginning of the Late Pleistocene.

Despite substantial progress over the past decade, the Paleolithic record of Arabia still features significant gaps (Fig. 4). These are most obvious during the supposedly arid MIS 6, 4 and 2. The presented Jebel Faya data now begin filling the early MIS 6 gap. With this, our results question the suitability of the glacial-interglacial demographic model and suggest the application of regionally-resolved climatic and ecological data (see^53^ for a similar conclusion in an African context), alongside data such as the IOM Index for the contextualisation of archaeological records. Given the climatic, ecological and geomorphic heterogeneity of Arabia, we also suggest that the notion of ‘windows’ is somewhat dichotomous and may be too simplistic a framework for understanding the complexities of ancient demography. Our results demonstrate that improving our understanding of the early prehistory of Arabia and related anthropological research questions requires targeting the identified chronological gaps through continuous interdisciplinary field work. Concluding human absence from presumed climatic and ecological conditions can be misleading. The search for fresh evidence that further fills in gaps in the archaeological record of Arabia may require working in less well studied geographic settings. These recent developments demonstrate that Arabia has not yet disclosed all secrets about its early settlers.

## Materials and methods

### Archaeological excavations

Excavations were conducted using quarter meter units within defined trenches. We piece-plotted all lithic artifacts larger than 2 cm and each bucket of sediment removed from the site using a Leica Total Station. To maximize recovery of small finds we dry screened all buckets at the site through 6 and 3 mm mesh and floated selected sediment samples at the field lab. To gain more detailed information about site taphonomy and vertical distribution of finds, we defined archaeological (AH) and geological horizons (GH) during excavation. To distinguish GHs and AHs we used Arabic numerals for geological layers and Roman numerals for archaeological layers. Numbers increase from top to bottom. Sediment characteristics were examined using micromorphological methods and sediment studies in the context of sedimentological research (see below). Analysis of the lithic assemblages followed standard procedures.

### Chronology

As the sediment consists mainly of coarse material (gravel to blocks), containing little quartz sand required for OSL dating, sampling was carried out at night using red headlights. The light-exposed outer layer of the exposure was first removed and large amounts of sediment were extracted and sieved on-site to remove the coarse fraction. The fine fraction (sand and silt) was transferred into opaque bags. Bulk sediment representing the sample surroundings was sampled at the same time for dosimetric measurements (high-resolution gamma spectrometry). In the laboratory, the quartz fraction (100–150 µm) was extracted and equivalent dose (D_e_) was determined using the standardised growth curve single-aliquot regenerative dose approach (1 mm aliquots). Most samples show a moderate spread of De values which calls for the application of different so-called age models (see supplemental material for details).

### Sediment analyses

Sediment samples were collected from the open trench face (Fig. 2) in zip lock bags. Loss-on-ignition (LOI) analyses followed the method described by Heiri et al.^54^. Results are reported as percentages of the dry weight. To determine grain size, samples of air-dried sediment < 2 mm were gently disaggregated in deionized water with 5% Calgon (Sodium hexametaphosphate) and analyzed using a Malvern Mastersizer 2000. Grain size statistics are based on the logarithmic graphical measures of the (original) Folk and Ward^55^ method for sorting, skewness and kurtosis. The mean particle size calculations are based on the modified Udden-Wentworth scale and are reported in microns (μm). Geochemical analysis was conducted using an Olympus Vanta pXRF.

## Supplementary Information

Supplementary Information.
