# Supplementary material for: Multiple phases of human occupation in Southeast Arabia between 210,000 and 120,000 years ago
Source: Sci Rep. 2022 Jan 31;12:1600. doi: 10.1038/s41598-022-05617-w (PMC8803878; doi:10.1038/s41598-022-05617-w)
Supplement: Supplementary file 1 — Supplementary Information. [file 41598_2022_5617_MOESM1_ESM.docx]

Supplementary Information for

**Multiple phases of human occupation in Southeast Arabia between 210,000 and 120,000 years ago.**

Bretzke, K.*, Preusser, F., Jasim, S., Miller, C., Preston, G., Raith, K., Underdown, S.J., Parton, A., Parker, A.G.

Corresponding author: Knut Bretzke, email: knut.bretzke@uni-jena.de

**The archaeological site FAY-NE1, Jebel Faya**

**Site description.** Jebel Faya is one of five mountains in a north-south oriented anticline structure composed of Upper Cretaceous limestone formations covering a basis of metamorphic rocks. Site FAY-NE1 is located at the northern end of Jebel Faya (Fig. S1). The area protected by the rock shelter is about 20 by 3.5 m (Fig. S2). Results reported here come from the Faya rock shelter sequence in the southwestern corner of the site. Excavated trenches cover areas under the present roof and the transition to the terrace in front of the rock shelter. The depositional environment is characterized by low energy processes including the erosion of the limestone and the deposition of small debris. The slope connecting the current rock shelter and the terrace is covered by large (*c.* 3 x 4 m) blocks that have formed the roof previously. To connect the shelter and the terrace sequence, we removed one of these blocks and recovered the lower part of the archaeological sequence below this block. The location of the archaeological remains clearly indicates that the former occupied area was located about 3 m east of the current rock shelter. Erosion of the limestone on the back of the shelter and the related falling of the roof created a stepwise relocation of the inhabitable area of the rock shelter to the west.

The rock shelter opens to the east, which enables the monitoring of the c. 20 km-wide plain stretching between Jebel Faya and the Hajar Mountains to the east. The plain inclines slightly and drains surface and underground water from the Hajar Mountains to the west. The anticline structure channels surface flow and forces underground water to the surface, which leads to an increased fresh water availability along the anticline and the formation of springs as observed at Jebel Buhais (1). These hydrological characteristics in addition to an ecotone setting composed of mountains, the plain and the desert to the west, created remarkable conditions that have repeatedly attracted Pleistocene hunter-gatherer groups.

**Archaeological excavation and lithic assemblages.** The ~3 m deep rock shelter sequence of site FAY-NE1 was excavated to bedrock between 2009 and 2017, with additional sampling seasons in 2018 and 2019. This archaeological sequence contains in total 6,351 lithic artifacts, excavated from an area of 25 m². Stratigraphic integrity of the sequence was verified through piece-plotting of finds, systematically collected dating samples and supporting sediment studies including grain size analysis, geochemistry, and micromorphology.

Bretzke et al. (2) have presented the stratigraphic correlation of the rock shelter sequence and the terrace sequence, which was presented by Armitage et al. (3). They show that AH VI can be stratigraphically linked to Assemblage C, while Assemblage B correlates with AH V and Assemblage A with AH IV. The older AH VII from the rock shelter has currently no stratigraphic equivalent in the terrace sequence. This is due to differences in the depositional environment of both sequences. Sedimentological analyses show greater impact of high energy processes such as substantial surface water flow and removal of sediments on the terrace during periods of strong precipitation. The rock shelter sequence was protected by the rock and was not affected by water flow. The sediment body of the rock shelter sequence is mainly composed of small limestone fragments produced by the weathering of the rock and blown in sand (Figs. S3, S4 and see sediment analysis below). Conditions for the preservation of occupation events are thus much better in the rock shelter sequence. The identified depositional processes led to a composition of the excavated sediments that is characterized by a developed compactness, which hinders the movement of artifacts. This in addition to relatively sharp edges of the lithic artifacts led us to conclude that the archaeological layers are largely in-situ.

We focus here on the lower three archaeological layers AHs VII-V, which were excavated from an area of 17 m². Combined these three archaeological layers provide a total of 1533 lithic artifacts larger than 2 cm (Table S1). The lowermost layer defined during excavation is geological horizon 7 (GH 7), which is composed of reddish sediments containing subangular, medium to coarse gravel sized limestone clasts. GH 7 ranges in thickness between 23 and 41 cm and contains archaeological layer VII. GH 7 features in its upper part a number of larger clasts of 30 to 45 cm in size forming a well-defined border to the overlying layer GH 6. GH 6 is relatively thick (~1.2 m) and composed of reddish-brown fine sand with some silt and medium sand. Within GH 6, the degree of carbonate cementation increases significantly with increasing depth (Fig. S4) however, there are few other signs of diagenesis. GH 6 contains archaeological horizons AH VI and AH V.

The typological composition of the lithic assemblages from AHs VII-V shows remarkable similarities, including the dominance of rather simple tools such as scraper as well as denticulated and notched artifacts (Table S2). While saying this, AH V features distinct characteristics such as the lack of bifacial artifacts, an increased proportion of pointed artifacts and the occurrence of inverse retouch. However, flake production from Levallois cores dominates in all archaeological layers. Unidirectional and centripetal reduction dominate in layers AH VII and VI, while AH V shows an increased number of cores featuring convergent scars at the expanse of centripetal reduction. Bretzke et al. (2) have already pointed out that the increase in convergent morphologies observed in AH V is not reflected in the occurrence of typical unidirectional convergent Levallois methods. Given broad and overlapping technological and typological spectra in all of the three presented AHs, we argue that the observed diachronic patterns in the lithic material culture reflect shifting preferences within a range of shared approaches rather than the introduction of something significantly new.

**Sediment analysis.**

**Grain size and geochemistry.** Four main sedimentary facies have been identified in the Faya Tr38N (Fig. S4) sequence noting that there is some lateral variation in the thickness of facies and size of clastic material. Two of these facies are characterized by sediment derived from the rock-shelter environment. These are Facies 1, RD – rockfall deposits and Facies 2, FO – fine-grained infiltrated deposits. RD is further subdivided into RD1, clast supported, unsorted, angular gravel to boulder sized debris, which is slightly weathered. These clasts are derived from the Cretaceous Simsima Fm limestone that forms the rock shelter vault and represent breakdown deposits from block collapse (sensu 4). RD2 comprises fine- to medium-grained sediment with a compositional affinity to the creamy yellowish-brown, fine-grained wackestone and/or lime mudstone, of the host rock derived by the weathering of the host rock. This process can be seen today on the exposed back wall of the rock shelter. Facies 3, AE – aeolian deposits are derived from externally derived processes typical of the surrounding landscape. These sands have an orange hue due to an increase in the percentage of oxidized quartz grains. To the west of Jebel Faya the composition of the sands is approximately 67% carbonate, 30% quartz and 3% Fe-Mg-rich grains (5). Facies 4, WL – water lain comprises sediment which has been reworked by water comprising small gravel and debris similar in size to clasts of underlying and overlying beds, with an open framework texture, where the matrix is absent.

Sediments were placed in a drying oven for 12 hours at 105°C and disaggregated using a rubber pestle and sieved using a 2 mm aperture sieve to remove any clasts. Samples were placed in beakers and dispersed in deionized water and organics removed using a few drops of 30% H_2_O_2_ and heated gently on a warming plate until reaction ceased. Any excess peroxide was evaporated until dry. Samples were disaggregated using deionised water with the addition of 10ml of 5% sodium hexametaphosphate and placed on an orbital mixer for 1 hour. Grain size calculations were determined using a Malvern Mastersizer 2000 on the <2 mm fraction. Sediment statistics (mean, sorting, skewness, kurtosis) follow the scheme proposed by Folk and Ward (6).

The lowermost part of Tr38N comprises facies FO and contains lithic assemblage VII (Fig. S5). The sediment matrix comprises coarse silts and fine sands (mean phi 3.5-4.5) that are very poorly sorted, mesokurtic and finely skewed. Archaeological layer VII is capped by rockfall debris (facies RD) with clasts up to 30 - 45 cm across along the section with FO interstitial material. No lithics were observed in this part of the sequence. AH VI sediments are leptokurtic, very poorly sorted, near symmetrical to leptokurtic, fine sand (Figs. S6, S7). The sediment is cemented by carbonate, supported by the higher LOI 950°C (carbonate) and calcium (Ca) values. At 140-130 cm, directly above AH VI, a limestone block line, represents RD rockfall debris with interstitial FO (Figs. S4, S5). The carbonate cemented matrix comprises very poorly sorted, coarse skewed, leptokurtic to very leptokurtic, very fine sand (Figs S6, S7).

Both AH V and IV are characterized by near-symmetrical, leptokurtic fine sands associated with facies AE (aeolian) (Figs. S4, S5). AH IV is separated between 55-70 cm by a clast-rich dominated horizon WL facies with fewer fines, which represents sediment sorted by water. Rock fall debris (RD) occurs at 45 cm and infiltration FO facies occurs between 40 cm - 0 cm which has a higher silt content. Within this unit at 32-29 cm the WL facies with fewer fines is characteristic of a paleodrip line, representing a pre-collapse position of the rockshelter roof. These sediments are leptokurtic, poorly sorted and coarse skewed characterized by a fall in LOI550.

**Micromorphological analysis.** Block samples were cut from the profile face and stabilized in the field with plaster-of-paris bandages to facilitate transport back to the Geoarchaeology Laboratory at the University of Tübingen. Here they were oven dried at 40˚C and then indurated under vacuum using a 7:3 mixture of unpromoted polyester resin and styrene, with methylethylketone peroxide (MEKP) as the catalyzing agent. After several weeks the mixture achieved a gel-like consistency and they were heated for 24h at 40˚C to completely harden the resin. They were then sliced using a rock saw and cut into chips measuring 9x6cm. The chips were then thin sectioned to 30µm thickness, digitally scanned (7) and analyzed under plan polarized light, cross polarized light and oblique incident light using a Zeiss Axioimager petrographic microscope. Analysis and description of the thin sections followed protocols from Courty et al. (8) and Stoops (9).

The fine fraction of the deposits is dominated by calcareous, micritic grains of sand with common (ca. 20-30%) sand-sized grains of quartz or feldspar. These grains are sub-rounded, uniform in size, and appear aeolian in origin. Coarser grains, ranging from coarse sand to gravel, are frequent and consist of sub-angular clasts of fossiliferous limestone, likely derived from the breakdown of the shelter bedrock.

**OSL dating**

**Sampling.** The deposits investigated in this study represent mainly rather coarse material originating from roof collapse mixed with aeolian sand. The latter is rather diffusely distributed throughout the sediment and does not form pronounced layers of spots. The presence of large carbonate clasts prevents the use of metal tubes for sampling. This led us to carry out all sampling during night while using red-light headlights and torches. We first removed several centimeters (5-10 cm) of the sediment exposure to avoid any material that might have been affected by daylight after the excavation. The sediment extracted from the expose was sieved on-site (2 mm) to substantially reduce the amount of material for shipping to the laboratory in Freiburg (probably down to 10% of the initial material being extracted). Material for dose rate measurements (c. 200 g) was sampled in parallel to the material later used for D_e_ determination but without sieving. The advantage of this approach is that a representative measurement of the material surrounding the D_e_ sample can be made and that this approach also allows for the detection of radioactive disequilibria in the Uranium decay chain (cf. 10). Such disequilibria can occur in sediments rich in carbonate, in particular during later mobilization of carbonates in case by percolating water. A loss or gain of isotopes from the Uranium decay can produce significant age offsets (10), but was not investigated in previous studies.

**Sample preparation.** Samples were first wet-sieved (100-150 µm) and subsequently treated with 10% HCl to remove carbonates, which caused a massive reduction of the sample material. The remaining material was dried, followed by heavy liquid separation using LST Fast Float (sodium heteropolytungstates dissolved in water) with densities of 2.70 g cm^-3^ and 2.58 cm^-3^, respectively, to enrich the quartz fraction. After drying, this fraction was etched in 40% HF for 1 hour with subsequent >1 hour 10% HCL treatment to remove fluorites, after rinsing with water. The rinsed material was dried and grains were mounted on stainless steel discs with a 1 mm silicon oil stamp as adhesive. Such discs contain ca. 50 grains each according to both theoretical considerations (11) and optical inspection. Considering that only about 3% of all grains from Jebel Faya produce acceptable luminescence characteristics (mainly due to no of very low signal output; 3), this results in an average number of 1.35 grains per aliquot, which will dominate the signal. Hence, the small aliquot approach used here will be very close to a single-grain level.

**D_e_ determination.** All measurements were done on a Freiberg Instruments Smart device (12). The single aliquot regenerative dose (SAR) protocol was used (13) and preheating at 230°C for 10 s was identified in dose recovery test (using the full SAR cycles used for D_e_ determination) as appropriate procedure (average dose recovery ratio 1.03 ± 0.04, n = 24). The OSL decay curves are quite bright, dominated by the fast component and show no problem with regard to feldspar contamination. Applied here was a standardised growth curve (SGC) approach (14; 15) using the ‘Least-Square normalisation’ procedure introduced by Li et al. (16). Seven full SAR dose response curves were measured for the construction of the SGCs using a double-saturating exponential function (Fig. S8). In addition, for 40 aliquots a reduced SAR protocol (natural and second regenerative dose step, both test dose corrected) have been measured. The Least-Square normalised standardized dose response curves for all samples are given below. Of the total number of measured reduced SAR aliquots, a certain number had to be rejected due to low signal levels, the number of totally accepted aliquots is given in Table S4.

The D_e_ distributions of all samples are given as Abanico plots further below (Fig. S9). For all samples, average D_e_ was calculated using the Central Age Model (CAM) and both the Minimum Age Model (MAM) or Finite Mixture Model (FMM) were tested (cf. 17). For the latter, for three components values were computed using sigma_b values between 0.15 and 0.25, and the value showing the best fitting criteria (BIC) was used for further calculations. Only for sample FAYA-38S-OSL8, only the CAM was calculated as both other approaches appeared inappropriate considering the statistical parameters (overdispersion, skewness, kurtosis) frequently used in analyses of D_e_ distributions. The overdispersion observed for this sample is 0.20, hence, in the middle of the range of sigma_b values tested for MAM analyses. We regard this to strongly support the chosen approach.

The reasoning behind using the MAM and FMM is justified as there appear three different processes being involved that are responsible for the placing grains at the position encounter during sampling. The main source of input is through aeolian transport (as indicated by our sedimentological and micromorphological analysis), which will deposit grains that likely will be well-bleached. Indeed, two modern aeolian samples taken close to the site gave mean D_e_ values of 0.05 ± 0.04 Gy (n = 18; FAYA Modern 1) and -0.05 ± 0.05 Gy (n = 16; FAYA Modern 2). The aeolian fraction is expected to be dominant in the D_e_ distributions. A second source of grain input will be delivery of grains from weathering of the rock shelter roof, as already discussed by Armitage et al. (3) and confirmed by our own sedimentological and micromorphological analysis investigations. While the amount of quartz in the limestone is quite low, the volume of roof material that disintegrated with time is quite substantial. The OSL signal in such grains may not have been zeroed at the time of deposition and will hence likely produce a population of D_e_ values that are not related to the age of sediment deposition and, hence, the age of the archaeological layer. Such D_e_ values must not be considered when calculating the average amount of dose absorbed since burial and the most common approach to do so is the MAM. In addition, from the theoretical point of view, and confirmed by sedimentological observations on-site, post-depositional mixing, possibly bioturbation, may introduce grains with an OSL signal that reflect a young daylight exposure time than the formation of the archaeological layer. This has already been highlighted by Armitage et al. (3), who used the FMM in such case sample show a population of D_e_ values at the lower edge of the distribution. Indeed, we observe populations of grains with much lower D_e_ values in some samples and interpret these to represent post-sedimentary mixing.

**Dose rate and age calculation.** After shipping to Freiburg, the material was dried, grinded and measured at VKTA Rossendorf e.V. for the concentration of K, Th and U by high-resolution gamma spectrometry (cf. 18). The comparison of the activities determined for U-238 and Ra-226 reveals no indication for the presence of radioactive disequilibrium in any of the samples investigated. Water content was assumed at 3±3% considering the mainly arid conditions in the region. Dose rates and ages were calculated using ADELEv2017 (10; add-ideas.de), taking into account longitude, latitude and sample depth for cosmic dose rate (19). The dosimetric data and ages are compiled in STable 4.

**Discussion of age reliability.** The samples show general well-behaving OSL properties. The OSL signal itself appears unproblematic and the majority of D_e_ values is in the range of ca. 100 Gy and far below 2D0. We are hence not expecting problems with age underestimation (cf. 20). The application of the SGC approach also proved highly acceptable, with figures-of-merit (FOM) all below the 10% limit suggested by Peng and Li (21). For six of the eight samples, the FOM is actually below 5%. The D_e_ distributions show a moderate spread that might be related to some grains being partially bleaching (input from the rock shelter) and infiltration processes (post-depositional). However, at least some of the D_e_ spread might be explained by the low dose rate and the inhomogenous nature of the sampled sediments (cf. 22). The dominant population in the dose distribution is, also due to its good OSL properties, expected to come from grains of aeolian origin.


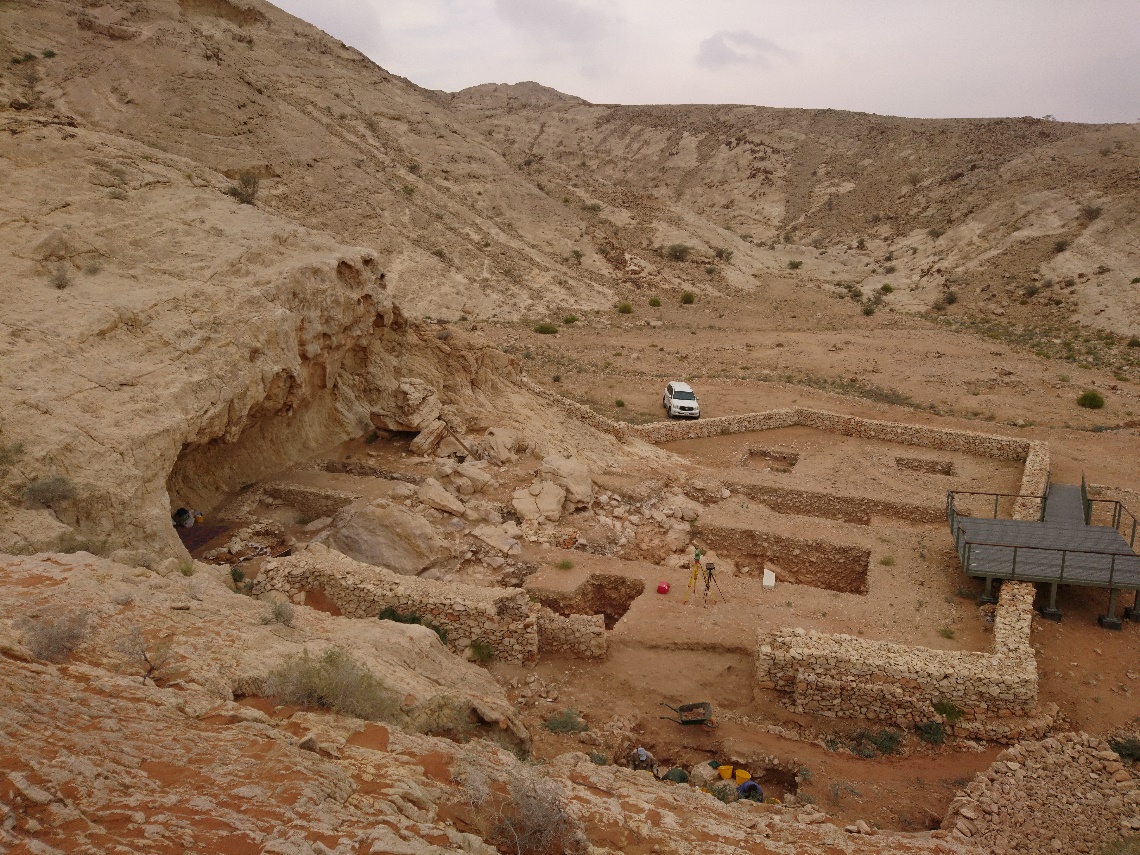


Fig. S1. Overview site FAY-NE1, Jebel Faya. View to north.

Fig. S2. Excavation plan. Red line shows the Faya Shelter excavation, dotted line in trench 38 shows the location of the profile shown in figure 2 in the main text. The OSL sample from Assemblage D was collected from the northern end of trench 27. Figure produced using QGIS 3.4. (URL: https://qgis.org) and Inkscape 1.0 (URL: https://inkscape.org).

**
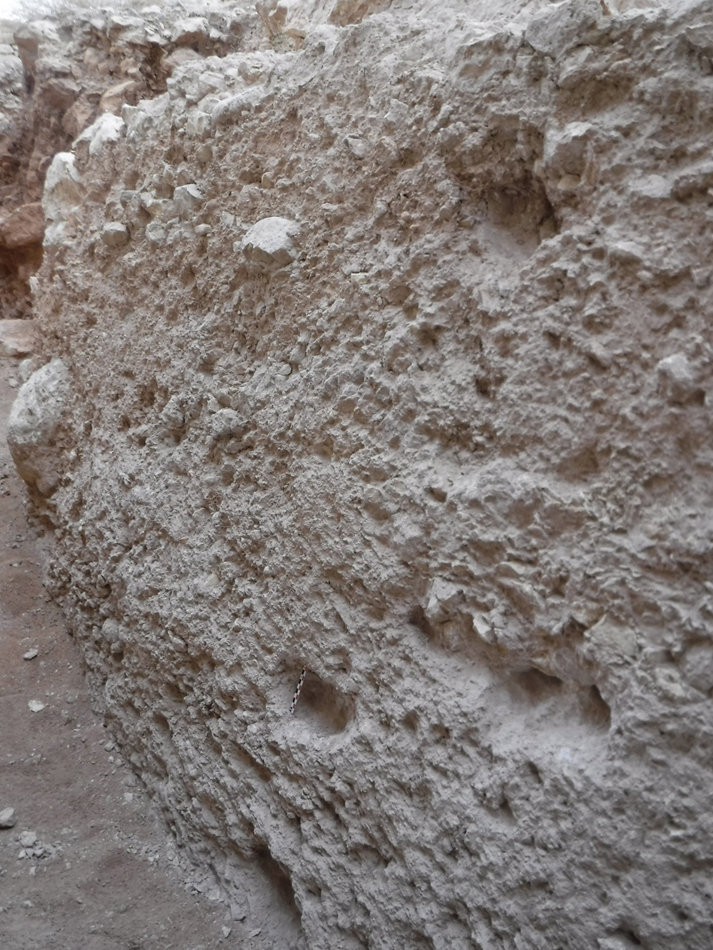
**

Fig. S3. Photo showing the southern profile of trench 38. Please note the mix of matrix and clasts throughout the sequence without macroscopically detectable layers.

**
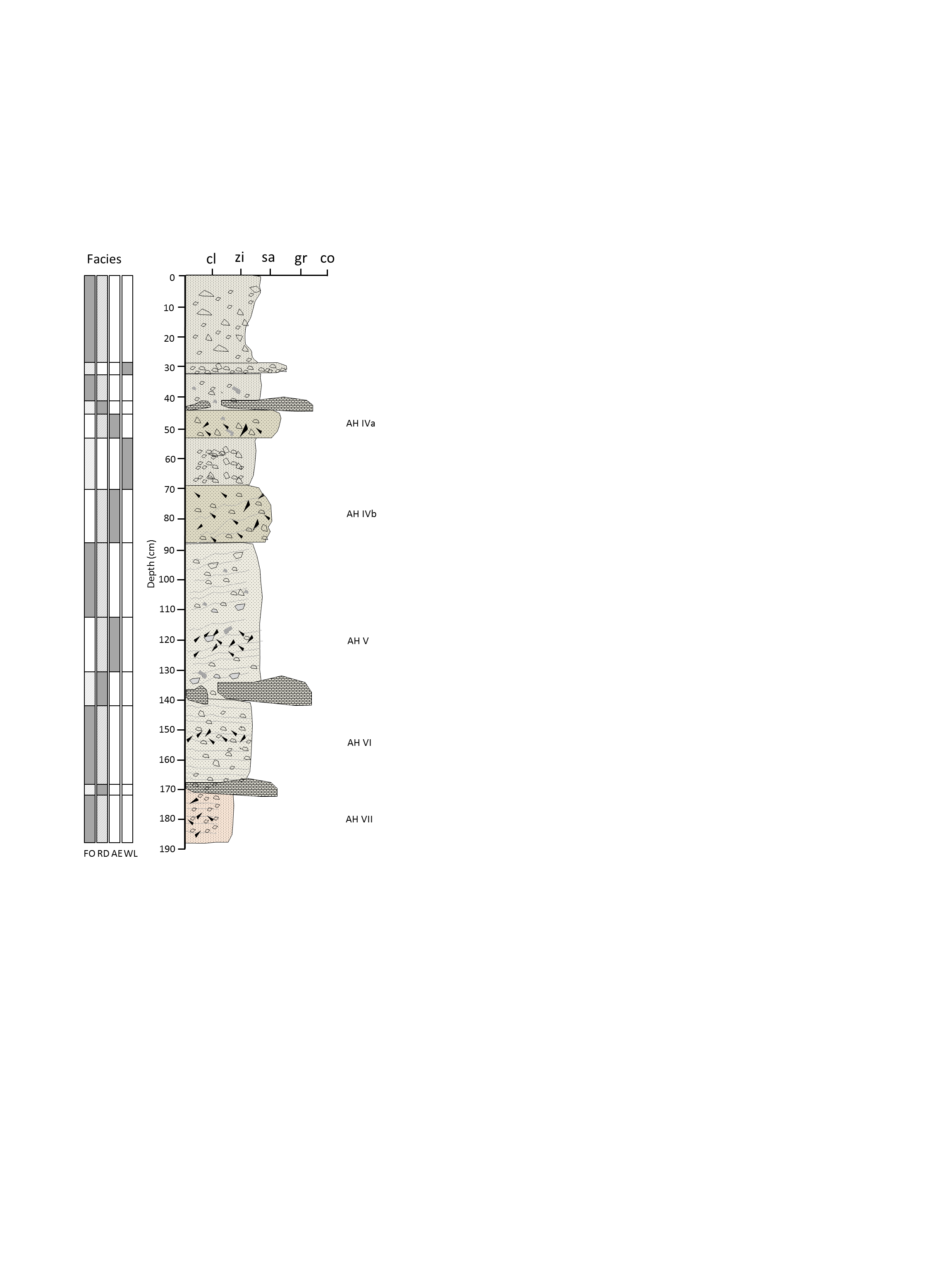
**

**Figure S4.** Sedimentary log from the Tr38 column section, with the main depositional processes represented for each bed and position of the archaeological layers (AHVII-IV).

**
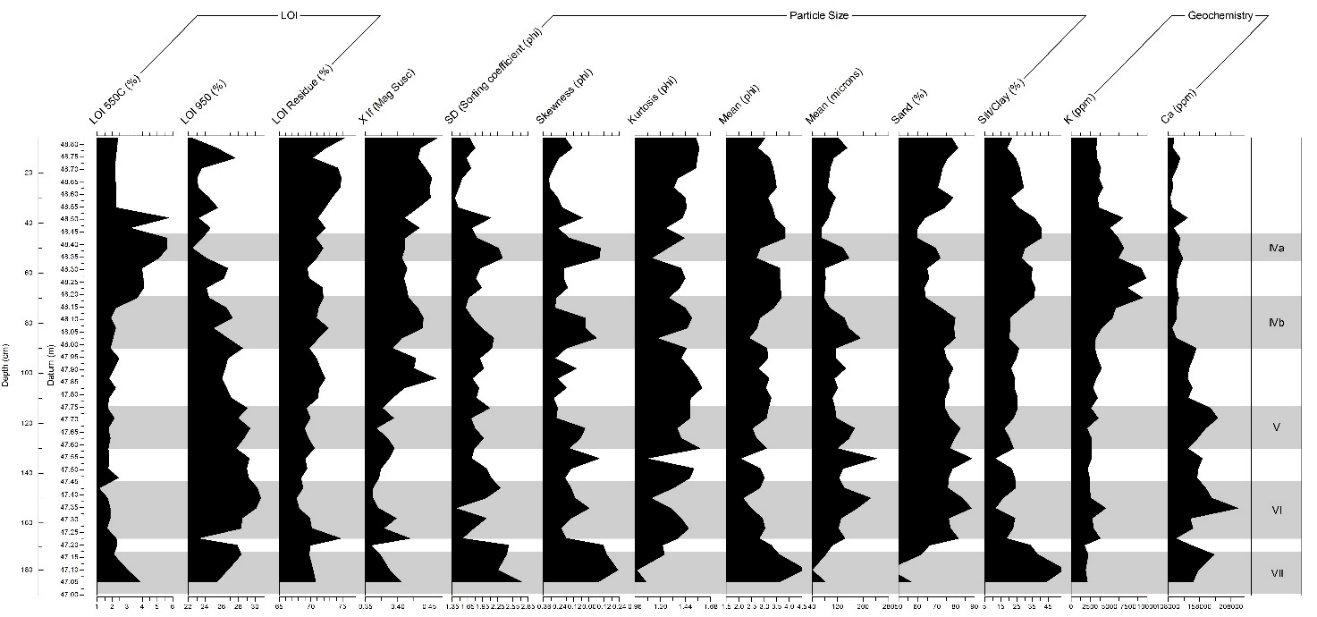
**

Fig. S5. Sediment properties from Tr38 section. Archaeological layers denoted by gray bars

Fig. S6. Biplot of skewness vs kurtosis for Tr38 sediments. Occupation levels shown in blue, non-archaeological levels in red.

Fig. S7. Biplot of sorting coefficient vs skewness for Tr38 sediments. Occupation levels shown in blue, non-archaeological levels in red.

FAYA19

FAYA-38S-OSL3

FAYA-38S-OSL4

FAYA-38S-OSL5

FAYA-38S-OSL6

FAYA-38S-OSL7

FAYA-38S-OSL8

FAYA-19N-OSL9

Fig. S8. Least-Square normalised standardized dose response curve of all samples presented here.


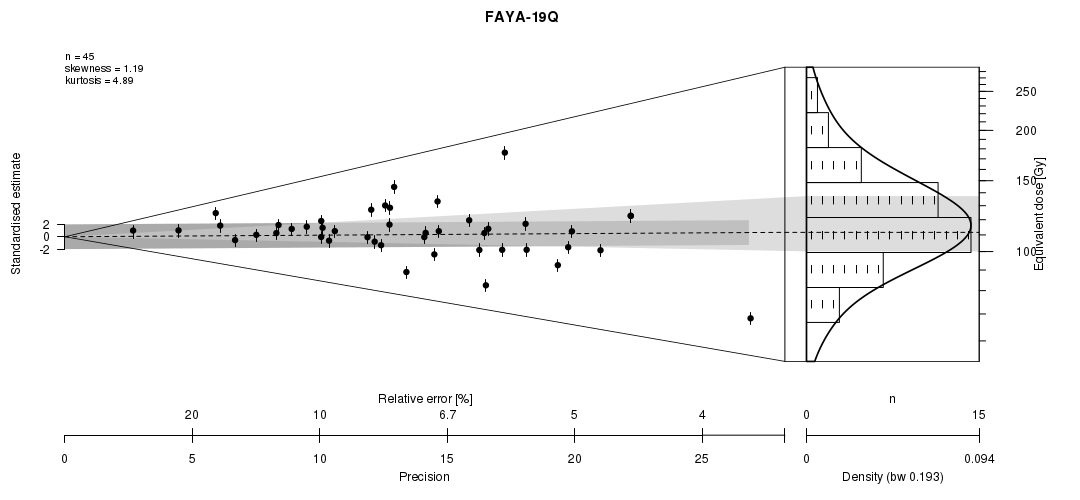


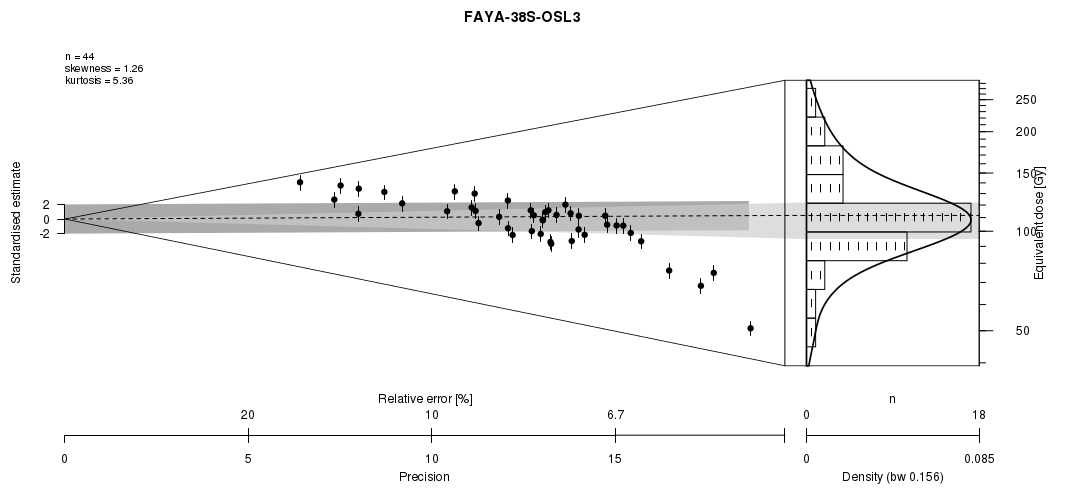


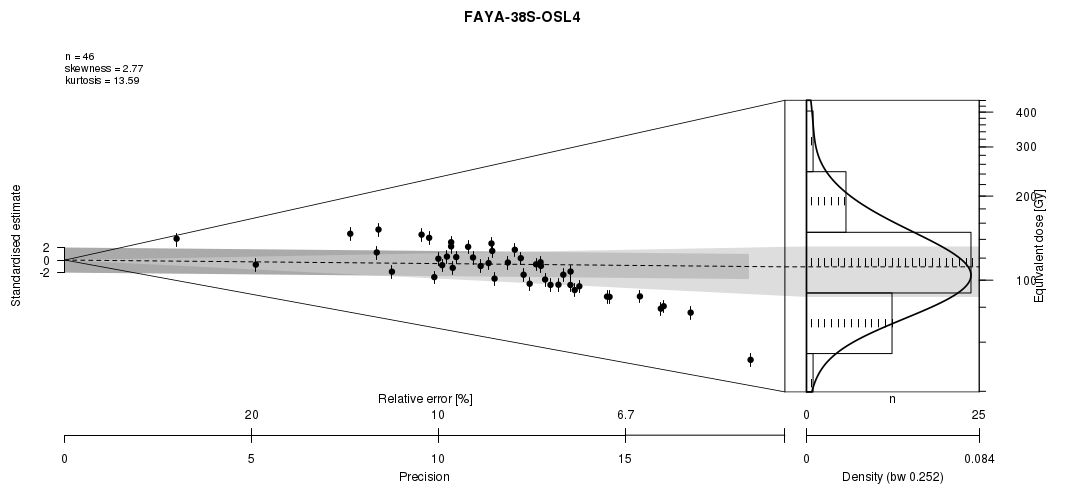


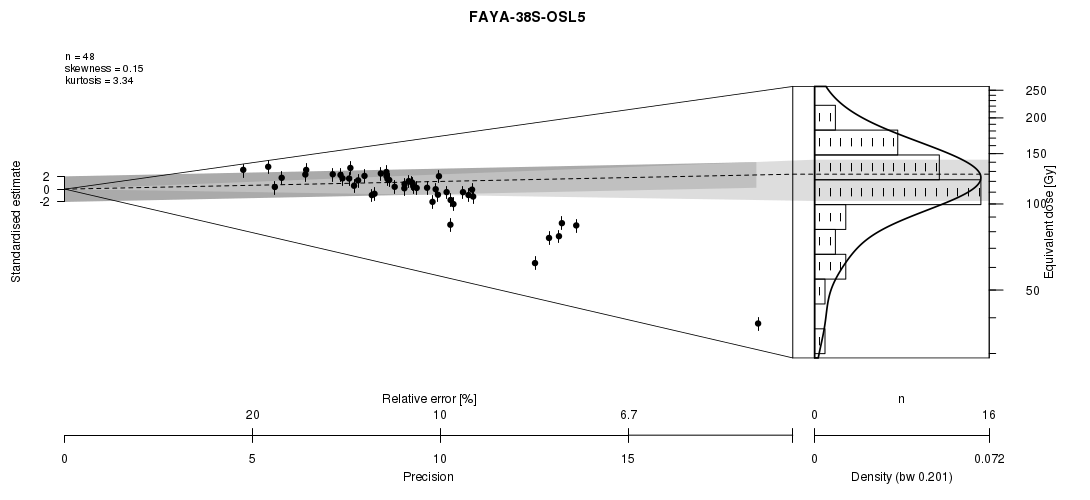


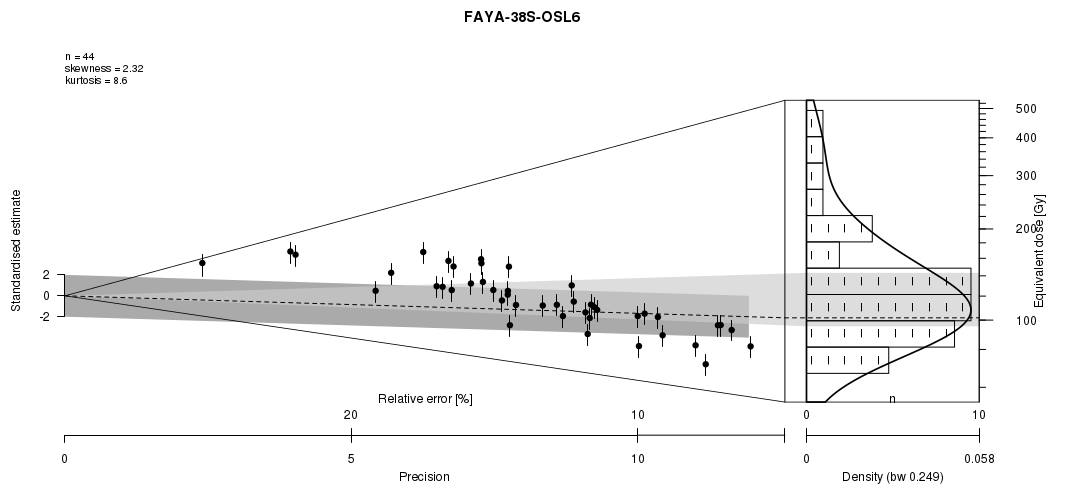


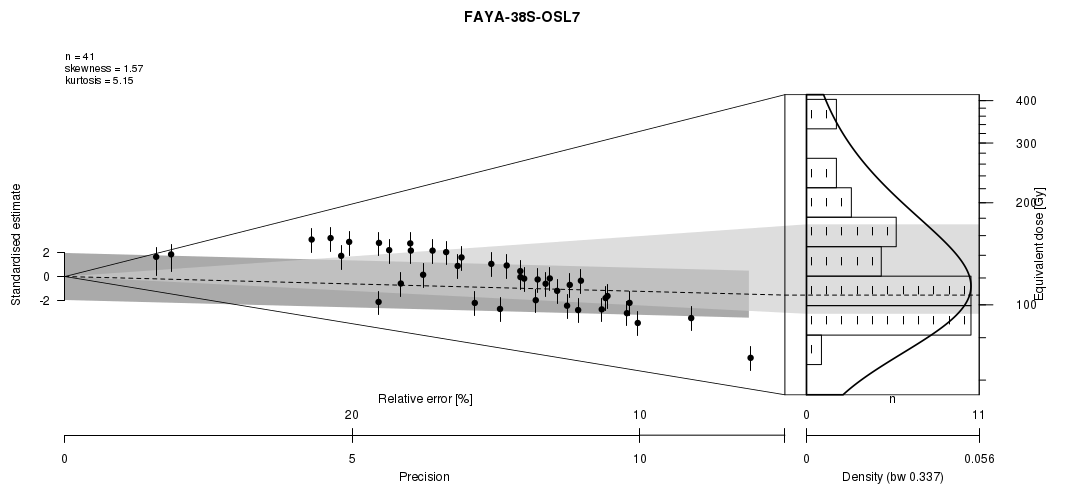


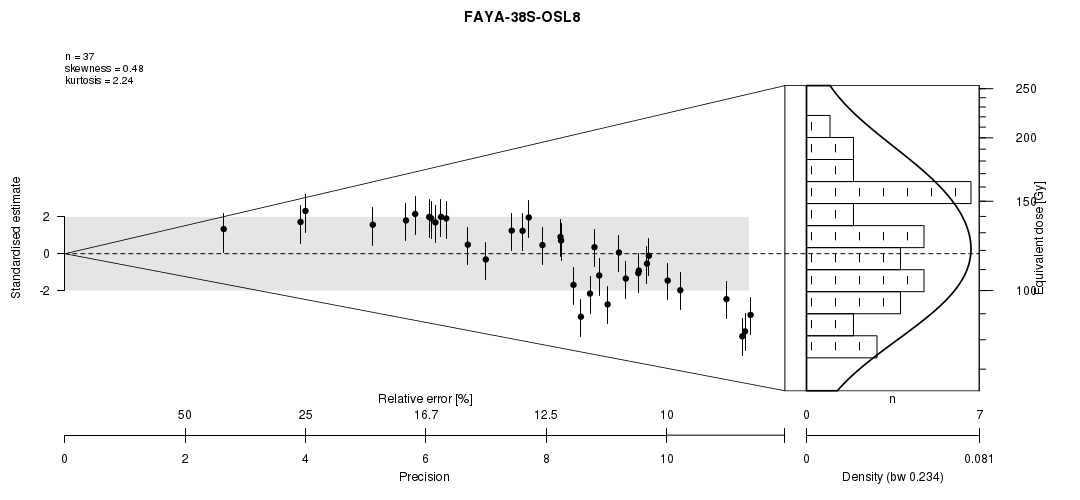


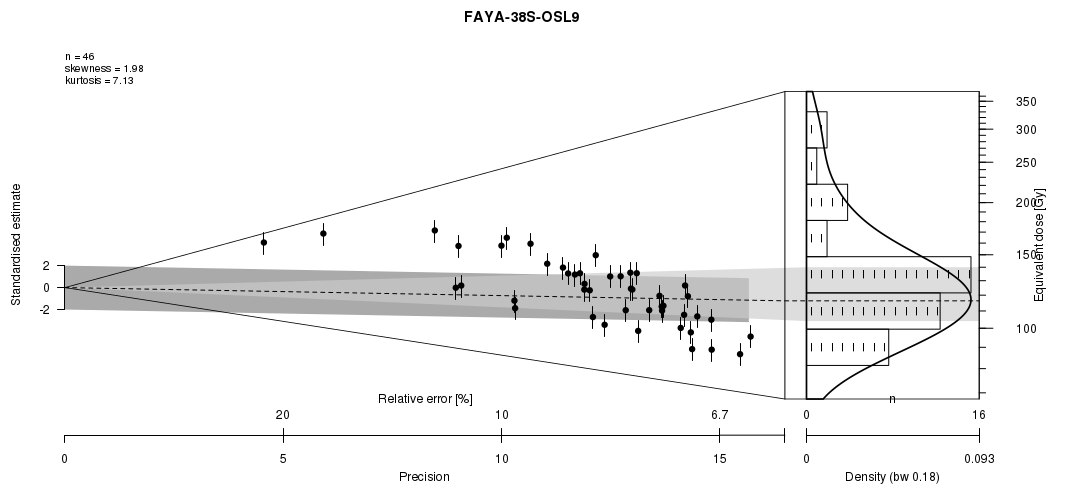


Fig. S9. Abanico plots for all samples presented in this study showing the individual De values together with the CAM range (light gray) and the mean D_e_ value used for age calculation (dark grex). See Dietze et al. (23) for details on plots. Plotted using the CLL Shiny Server: rlum.geographie.uni-koeln.de (author C. Burow).

Table S1. Basic characteristics of lithic assemblages.

| **AH** | **total number of artifacts** | **excavated area in m²** | **excavated volume in m³** | **artifacts/m³** | **debitage**  **n** | **cores**  **n** | **tools**  **n** |
| --- | --- | --- | --- | --- | --- | --- | --- |
| V | 755 | 17 | 5.1 | 148 | 703 | 20 | 32 |
| VI | 477 | 17 | 2.7 | 177 | 428 | 17 | 32 |
| VII | 301 | 17 | 1.8 | 167 | 275 | 7 | 19 |

Table S2. Proportion of tool types.

| **Tool type** | **V** | **VI** | **VII** |  |  |  |
| --- | --- | --- | --- | --- | --- | --- |
| Scraper | 42 % | 33 % | 47 % |  |  |  |
| Burin | 6 % | 2 % | 0 % |  |  |  |
| Point | 21 % | 4 % | 11 % |  |  |  |
| Bifacial | 0 % | 23 % | 5 % |  |  |  |
| Denticulate/Notch | 24 % | 29 % | 37 % |  |  |  |
| unsystematic retouch | 6 % | 9 % | 0 % |  |  |  |
| **Number total** | **32** | **32** | **19** |  |  |  |

Table S3. Proportion of core reduction systems.

| **Core reduction systems/scar patterns** | **V** | **VI** | **VII** |
| --- | --- | --- | --- |
| Flat hierarchical |  |  |  |
| Radial | 5 % | 29 % | 29 % |
| Orthogonal | 15 % | 7 % | 0 % |
| Unidirectional | 20 % | 24 % | 29 % |
| Convergent | 30 % | 6 % | 7 % |
| Bidirectional | 5 % | 2 % | 7 % |
| Tournant/semi-tournant | 5 % | 5 % | 7 % |
| Multiple platform | 0 % | 17 % | 21 % |
| Ventral core | 20 % | 0 % | 0 % |
| Indet | 0 % | 10 % | 0 % |
| **Number total** | **20** | **17** | **7** |

Table S4. Summary data of OSL dating with sample name, assigned archaeological horizon, sampling depth below surface, and activity of dose rate relevant elements (K, Th, U). For the latter, the activity of the initial (U-238) and final part (Ra-226) are given to allow checking for radioactive disequilibrium. D gives the total dose rate, n the number of aliquots accepted for De determination, od. = observed overdispersion, Model = dose model applied for calculation of average De, CAM = Central Age Model, FMM = Finite Mixture Model, MAM = Minimum Age Model.

| Sample | AH | Depth  (cm) | K  (Bq/kg) | Th  (Bq/kg) | U  [U-238]  (Bq/kg) | U  [Ra-226]  (Bq/kg) | D  (Gy ka^-1^) | n | od | Model | CAM  D_e_  (Gy) | Model  D_e_  (Gy) | CAM  Age  (ka) | Model  Age  (ka) |
| --- | --- | --- | --- | --- | --- | --- | --- | --- | --- | --- | --- | --- | --- | --- |
| FAYA-38S-OSL3 | V | 278 | 139±13 | 5.7±0.5 | 10.2±2.1 | 12.8±0.9 | 0.91±0.13 | 44 | 0.27 | FMM | 108.87±4.64 | 111.64±7.94 | 120±17 | **123±10** |
| FAYA19 | VI | 95 | 136±12 | 4.8±0.4 | 10.3±1.6 | 10.7±0.7 | 0.86±0.05 | 45 | 0.28 | FMM | 117.30±4.68 | 115.99±5.72 | 137±8 | **135±9** |
| FAYA-38S-OSL4 | VI | 300 | 108±12 | 4.1±0.3 | 8.4±1.4 | 9.8±0.7 | 0.72±0.06 | 47 | 0.44 | MAM | 104.34±6.79 | 98.98±8.71 | 144±12 | **137±14** |
| FAYA-38S-OSL7 | VI | 347 | 135±14 | 5.1±0.4 | 7.5±1.2 | 10.4±0.7 | 0.83±0.09 | 41 | 0.28 | MAM | 121.21±6.00 | 106.85±10.73 | 146±10 | **129±14** |
| FAYA-38S-OSL8 | VI | 368 | 127±14 | 4.5±0.4 | 9.8±1.8 | 10.9±0.8 | 0.80±0.05 | 37 | 0.20 | - | 118.21±4.71 | - | **148±9** | **-** |
| FAYA-38S-OSL5 | VII | 311 | 115±10 | 4.3±0.3 | 7.4±1.5 | 9.3±0.6 | 0.74±0.04 | 40 | 0.34 | FMM | 112.70±5.77 | 127.11±5.18 | 153±10 | **172±10** |
| FAYA-38S-OSL6 | VII | 330 | 83±9 | 3.1±0.3 | 6.7±1.4 | 8.0±0.6 | 0.59±0.04 | 44 | 0.32 | MAM | 120.30±6.21 | 101.75±9.48 | 204±14 | **172±18** |
| FAYA-19N-OSL9 | D | 290 | 67±8 | 2.9±0.3 | 5.9±1.2 | 8.0±0.6 | 0.55±0.05 | 46 | 0.24 | MAM | 125.15±4.79 | 116.32±8.54 | 228±22 | **212±19** |

**SI References**

1. S. Holzkämper, A. Mangini, U/Th dating of carbonate precipitates at the excavation site of Jebel al-Buhais, in The natural environment of Jebel al-Buhais: Past and Present*,* H. P. Uerpmann, M. Uerpmann, S. A. Jasim, Eds. (Kerns Verlag, Tübingen, 2008), pp. 47-52.

2. K. Bretzke, N. J. Conard, H.-P. Uerpmann, Excavations at Jebel Faya – The FAY-NE1 Shelter Sequence. *Proceedings of the Seminar for Arabian Studies* **44**, 69-82 (2014).

3. S. J. Armitage *et al.*, The Southern Route “Out of Africa”: Evidence for an Early Expansion of Modern Humans into Arabia. *Science* **331**, 453-456 (2011).

4. W. B. White, Cave sediments and paleoclimate. *Journal of Cave and Karst Studies* **69**, 76–93 (2007).

5. A. R. Farrant *et al*., *Geology of the Al Dhaid 1: 100 000 Map Sheet*, 100-2. (United Arab Emirates, pp.1-61, 2006).

6. R. L. Folk, W. C. Ward. 1957. Brazos River bar: a study in the significance of grain size parameters. Journal of Sedimentary Petrology 27: 3–26

7. T. L. Arpin, C. Mallol, P. Goldberg, Short contribution: A new method of analyzing and documenting micromorphological thin sections using flatbed scanners: Applications in geoarchaeological studies. *Geoarchaeology: An International Journal* **17**, 305-313 (2002).

8. M.-A. Courty, P. Goldberg, R. I. Macphail, *Soils and Micromorphology in Archaeology* (Cambridge University Press, Cambridge, 1989).

9. G. Stoops, Guidelines for Analysis and Description of Soil and Regolith Thin Sections. (Soil Science Society of America, Inc, Madison, Wisconsin, 2003).

10. D. Degering, A. Degering, Change is the only constant - time-dependent dose rates in luminescence dating. *Quaternary Geochronology* **58**, 101074 (2020).

11. A. J. Heer, G. Adamiec, P. Moska. How many grains are there on a single aliquot? *Ancient TL* **30**, 9-16 (2012).

12. D. Richter, A. Richter, K. Dornich, Lexsyg smart — a luminescence detection system for dosimetry, material research and dating application. *Geochronometria* **42**, 202–209 (2015).

13. A. S. Murray, A. G. Wintle, Luminescence dating of quartz using an improved single-aliquot regenerative-dose protocol. *Radiation Measurements* **32**, 57-73 (2000).

14. H. M. Roberts, G. A. T. Duller, Standardised growth curves for optical dating of sediment using multiple-grain aliquots. *Radiation Measurements* **38**, 241–252 (2004).

15. B. Li, R. G. Roberts, Z. Jacobs, S.H Li. Potential of establishing a ‘global standardised growth curve’ (gSGC) for optical dating of quartz from sediments. *Quaternary Geochronology* **27**, 94-104 (2015).

16. B. Li, Z. Jacobs, R. G. Roberts, Investigation of the applicability of standardised growth curves for OSL dating of quartz from Haua Fteah cave, Libya. *Quaternary Geochronology* **35**, 1-15 (2016).

17. R. F. Galbraith, R. G. Roberts, Statistical aspects of equivalent dose and error calculation and display in OSL dating: An overview and some recommendations. *Quaternary Geochronology* **11**, 1-27 (2012).

18. F. Preusser, H. U. Kasper, Comparison of dose rate determination using high-resolution gamma spectrometry and inductively coupled plasma-mass spectrometry. *Ancient TL* **19**, 19–23 (2001).

19. J. R. Prescott, J. T. Hutton, Cosmic ray contributions to dose rates for luminescence and ESR dating: Large depths and long-term time variations. *Radiation Measurements* **23**, 497-500 (1994).

20. Wintle, A. G. & Murray, A. S. A review of quartz optically stimulated luminescence characteristics and their relevance in single-aliquot regeneration dating protocols. Radiation Measurements 41, 369-391 (2006).

21. J. Peng, B. Li, Single-aliquot Regenerative-Dose (SAR) and Standardised Growth Curve (SGC) Equivalent Dose Determination in a Batch Model Using the R Package ‘numOSl’. *Ancient TL* **35**, 32-53 (2018).

22. Y. S. Mayya, *et al.*, Towards quantifying beta microdosimetric effects in single-grain quartz dose distribution. *Radiation Measurements* **41**, 1032-1039 (2006).

23. M. Dietze *et al.*, The abanico plot: Visualising chronometric data with individual standard errors. *Quaternary Geochronology* **31**, 12-18 (2016).
